# Sleep disturbance increases the risk of severity and acquisition of COVID-19: a systematic review and meta-analysis

**DOI:** 10.1186/s40001-023-01415-w

**Published:** 2023-10-18

**Authors:** Arman Shafiee, Kyana Jafarabady, Shahryar Rajai, Ida Mohammadi, Sayed-Hamidreza Mozhgani

**Affiliations:** 1 Department of Psychiatry and Mental Health, Alborz University of Medical Sciences, Karaj, Iran; 2 Student Research Committee, School of Medicine, Alborz University of Medical Sciences, Karaj, Iran; 3 School of Medicine, Shahid Beheshti University of Medical Sciences, Tehran, Iran; 4 Department of Microbiology, School of Medicine, Alborz University of Medical Sciences, Karaj, Iran; 5 Non-Communicable Diseases Research Center, Alborz University of Medical, Sciences, Karaj, Iran

**Keywords:** SARS-CoV-2, COVID-19, Sleep, Sleep disorder, Treatment

## Abstract

**Background:**

Understanding the association between sleep quality and COVID-19 outcomes is crucial for effective preventive strategies and patient management. This systematic review aims to evaluate the impact of sleep quality as a risk factor for acquiring COVID-19 infection and the severity of the disease.

**Methods:**

A comprehensive search of electronic databases was conducted to identify relevant studies published from the inception of the COVID-19 pandemic which was 31^st^ of December 2019 until 30 April 2023. Studies investigating the relationship between sleep quality and COVID-19 infection, or disease severity were included. Random effect meta-analysis was performed with odds ratios (OR) and their 95% confidence intervals (95% CI) as effect measures.

**Results:**

Out of the initial 1,132 articles identified, 12 studies met the inclusion criteria. All studies were observational studies (cohort, case–control, and cross-sectional). The association between sleep quality and COVID-19 infection risk was examined in 6 studies, The results of our meta-analysis showed that participants with poor sleep quality showed a 16% increase regarding the risk of COVID-19 acquisition (OR 1.16; 95% CI 1.03, 1.32; *I*^2^ = 65.2%, *p* = 0.02). Our results showed that participants with poor sleep quality showed a 51% increase in the incidence of primary composite outcome (OR 1.51; 95% CI 1.25, 1.81; *I*^2^ = 57.85%, *p* < 0.001). The result of our subgroup analysis also showed significantly increased risk of mortality (RR 0.67; 95% CI 0.50, 0.90; *I*^2^ = 31%, *p* = 0.008), and disease severity (OR 1.47; 95% CI 1.19, 1.80; *I*^2^ = 3.21%, *p* < 0.001) when comparing poor sleep group to those with good sleep quality.

**Conclusion:**

This study highlights a significant association between poor sleep quality and an increased risk of COVID-19 infection as well as worse disease clinical outcomes.

**Supplementary Information:**

The online version contains supplementary material available at 10.1186/s40001-023-01415-w.

## Introduction

The COVID-19 pandemic caused by the novel coronavirus SARS-CoV-2 has had a profound impact on global health, with millions of individuals worldwide affected by the disease [1]. As the pandemic continues to evolve, there is a growing need to understand the factors that contribute to the risk of COVID-19 infection and the severity of the disease [2]. Sleep quality, a fundamental aspect of overall health, has been increasingly recognized as a potential risk factor for various health outcomes [3]. Exploring the impact of sleep quality on COVID-19 infection risk and disease severity is crucial for identifying modifiable factors that could inform preventive strategies and improve patient outcomes [4].

Sleep plays a vital role in maintaining optimal immune function, cognitive performance, and overall well-being [5, 6]. Adequate sleep is essential for the proper functioning of the immune system, including the production and regulation of immune cells and cytokines that defend against viral infections [6]. Disruptions in sleep patterns, such as insufficient sleep duration, poor sleep quality, and sleep disorders, have been associated with increased susceptibility to viral infections and decreased immune response [7, 8].

Given the significant interplay between sleep and immune function, it is plausible that sleep quality may also influence the risk of COVID-19 infection and the severity of the disease [8]. Sleep disturbances can contribute to immune dysregulation, impairing the body’s ability to mount an effective defense against viral pathogens [7, 9]. Moreover, poor sleep quality has been linked to chronic inflammation and underlying health conditions, which are known risk factors for severe COVID-19 outcomes [10].

Understanding the relationship between sleep quality and COVID-19 infection risk as well as disease severity is of paramount importance in managing the pandemic. By identifying sleep quality as a potential risk factor, public health interventions could be developed to promote healthy sleep practices and improve immune function, ultimately reducing the risk of COVID-19 infection and mitigating disease severity [10]. To address this research gap, we conducted a systematic review to comprehensively examine the impact of sleep quality on COVID-19 infection risk and disease severity.

## Methods

### Research question and objectives

The research question for this systematic review is: What is the impact of sleep quality as a risk factor for COVID-19 infection and disease severity?

### Study design

This systematic review followed the Preferred Reporting Items for Systematic Reviews and Meta-Analyses (PRISMA) guidelines to ensure a rigorous and transparent methodology [11]. Our study protocol is registered at PROSPERO under the number CRD42023426325.

### Search strategy

A comprehensive search of electronic databases was conducted to identify relevant studies. The databases searched included Medline (through PubMed), Scopus, Embase, medRvix, Cochrane Library, Google Scholar, and Web of Science. The search was conducted from the inception of the COVID-19 pandemic which is reported as 31st of December 2019 to 30 April 2023. The following search terms and their combinations were used: "COVID-19" OR "SARS-CoV-2" AND "sleep" OR "sleep quality" OR "sleep disturbance". The full search strategy is available in the Additional file 1. Additional studies were identified by manually searching the reference lists of included articles and relevant review papers.

### Eligibility criteria

The following inclusion criteria were applied: Studies that investigated the relationship between sleep quality and COVID-19 infection risk or disease severity; observational studies (e.g., cohort studies, case–control studies, cross-sectional studies) and interventional studies (e.g., randomized controlled trials) were included; and studies conducted on human participants.

The following exclusion criteria were applied: studies that did not assess sleep quality as an exposure or risk factor; animal studies, case reports, letters to the editor, and conference abstracts.

### Study selection and data extraction

Two independent reviewers screened the titles and abstracts of the identified articles for eligibility. Full-text articles were retrieved for potentially relevant studies. Any discrepancies were resolved through discussion and consensus.

A standardized data extraction form was developed and used to extract relevant information from the included studies. The following data were extracted: Study characteristics: author(s), publication year, study design, country; participant characteristics: sample size, age, sex, and population characteristics; and sleep quality assessment: measurement tools, definitions, and categorizations of sleep quality.

### Quality assessment

The methodological quality and risk of bias of the included studies were independently assessed by two reviewers using appropriate tools. The Newcastle–Ottawa Scale (NOS) was used for assessing the quality of both the observational studies [12]. Any discrepancies were resolved through discussion and consensus.

### Data synthesis and analysis

We utilized a random-effects meta-analysis approach, employing the generic inverse variance method, to combine the effect sizes from each study. We used StataCorp. 2015. Stata Statistical Software: Release 14. College Station, TX: StataCorp LP. to conduct random-effects meta-analyses with odds ratios (OR) and their 95% confidence intervals (95% CI) as effect measures. The assessment of heterogeneity was performed using the Cochran Q statistic and I2 value, where an I2 value of less than 35% indicated a low amount of heterogeneity [13, 14]. We performed a sensitivity analysis using leave one out method to investigate the robustness of our findings. Due to the limited number of included studies (*n* < 10), we did not conduct a funnel plot or perform an Egger regression test.

## Results

### Study selection

**Fig. 1:**
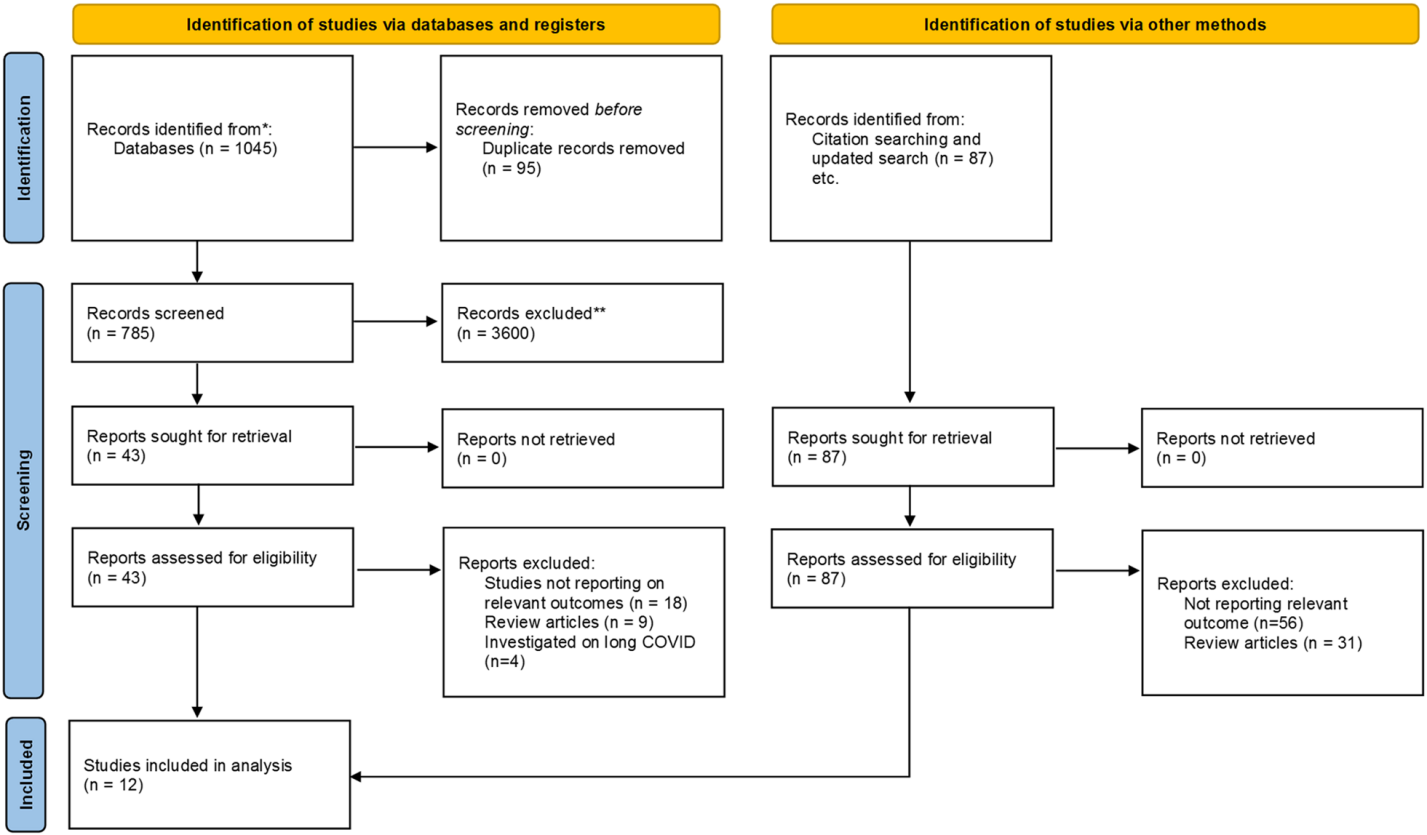
PRISMA flow-diagram

A total of 1,132 articles were identified through the initial database search. After removing duplicates, 785 unique articles remained. Titles and abstracts were screened, resulting in the exclusion of 728 articles that did not meet the eligibility criteria. The full-text assessment was conducted on the remaining 57 articles, of which 12 studies met the inclusion criteria. Figure 1 presents the PRISMA flowchart illustrating the study selection process.

### Study characteristics and quality assessments

The 12 included studies were all observational [15–27]. The studies were conducted in various countries, including the United States (*n* = 4), China (*n* = 2), the UK (*n* = 3), and one study conducted in Bangladesh, Poland, and Netherlands. Among the included studies, 6 studies evaluated the effect of poor sleep quality on COVID-19 severity [15, 20, 22, 23, 25, 26], and the remaining studies evaluated the effect of poor sleep quality as a risk factor of acquisition of COVID-19 [16–19, 21, 24]. Table 1 summarizes the characteristics of the included studies.

**Table 1:** Characteristics of the included studies

Author	Year	Country	Type of study	Sample size	Study population	Age	Male (%)	Sleep quality measure
Ahmadi [15]	2021	UK	Cohort study	468,569	Adults aged between 40 and 69 years from the UK Biobank	56.5 ± 8.1	45.40%	5-point scale based on: Morning chronotype, sleep duration (7–9 h), not usually insomnia, no snoring, and no frequent daytime sleepiness Poor sleep = 0 or 1 moderate sleep = 2 or 3 good sleeps = 4 or 5
Cloosterman [16]	2021	Netherlands	Cohort study	2586	Runners participating in an ongoing randomized controlled trial on running injury prevention among recreational runners	44.4 (12.2)	62	On five option scale for sleep disruption from 5 (strongly agree) to 1 (strongly disagree), sleep disruption was categorized as an answer of agree or strongly agree (4 or 5 points)
Elise [17]	2022	UK	Panel study (Longitudinal study)	1811	Adults from the UCL COVID-19 Social Study who had previously been infected with COVID-19	45–59 = 40.70% 30–44 = 23.91% 18–29 = 5.96%	24.41	5 option scale from very good, good, average, not good, and very poor Good sleep was categorized as very good/good, average sleep as average and poor sleep as not good/very poor
Gao [18]	2020	China	Case–control	105 cases and 210 controls	Patients with SARS-CoV-2 infection as the case group from the Wuhan Tongji Hospital, and 2 controls for each case from communities in Wuhan	54.3 (55 for case and 54 for control)	45.70%	Lack of sleep referred to sleep duration < 7 h per night. (Sleep duration = (5 × weekday sleep duration) + (2 × weekend sleep duration)/7)
Hayley [19]	2021	UK	Cohort study	15,227	Age 16 years or more and residence in the UK at the point of enrolment, recruited via a national media campaign	59.4 ± 13.4	30.2	Online questions asking about sleep hours
Huang [20]	2020	China	Cohort study	164	A history of SARS-CoV-2 infection confirmed by high-throughput sequencing or positive real-time reverse-transcription polymerase-chain-reaction, Chinese race, and age ≥ 18 years and discharged from one of 4 clinical centers in 3 provinces	44	50	Sleep status was defined according to national sleep foundation guidelines
Hyunju [21]	2021	USA	Case–control	568 COVID-19 cases and 2316 controls	Healthcare workers in 6 countries with a high frequency of workplace exposure to covid-19	48	71.6	The following 3 sleep problems were defined: (1) Did you have difficulties falling asleep at night? (2) Did you often wake up in the early hours, unable to get back to sleep? (3) Did you take sleeping pills more than 3 times per week? A score between 0 and 3 was given based on having these sleep problems
Jones et al. [22]	2022	USA	Cohort study	557,000	Individuals in the FInnGen database	N.R	N.R	ICD10-based electronic health record and questionnaire-based information on self-reported short sleep and insomnia and diagnosis of insomnia
Li et al. [23]	2021	USA	Cohort study	46,535	UK biobank	69.4 ± 8.3 years	46.70%	Sleep behavior burdens: “none” (0), “mild” (1), “moderate” (2–3), “significant” (4–6)
Marcus et al. [24]	2021	USA	Cohort study	14,335	English speaking adults with a smartphone	18–29 years: 1961 (13.7%) 30–39 years: 3225 (22.5%) 40–49 years: 2873 (20.0%) 50–59 years: 2839 (19.8%) + 60 years: 3437 (24.0%)	35%	Sleep duration
Mohsin et al. [25]	2021	Bangladesh	Comparative cross-sectional	1500	COVID-19 patients in Dhaka city	43.23 ± 15.48 years	69.20%	History of sleep disturbances
Pływaczewska et al. [26]	2022	Poland	Cohort study	1847	Participants of the STOP-COVID registry of the PoLoCOV-Study	Median age 51	34.50%	History of insomnia (defined as a difficulty falling asleep and maintaining sleep continuity during 4 weeks before COVID-19; falling asleep after midnight and nightshift work)

The methodological quality and risk of bias assessment revealed that the included studies exhibited varying levels of quality. The observational studies were generally assessed using the Newcastle–Ottawa Scale (NOS), with scores ranging from 5 to 9 (out of 9). There was no study with a high risk of bias (Additional file 1: Fig S1).

### Sleep quality and COVID-19 infection risk

**Fig. 2:**
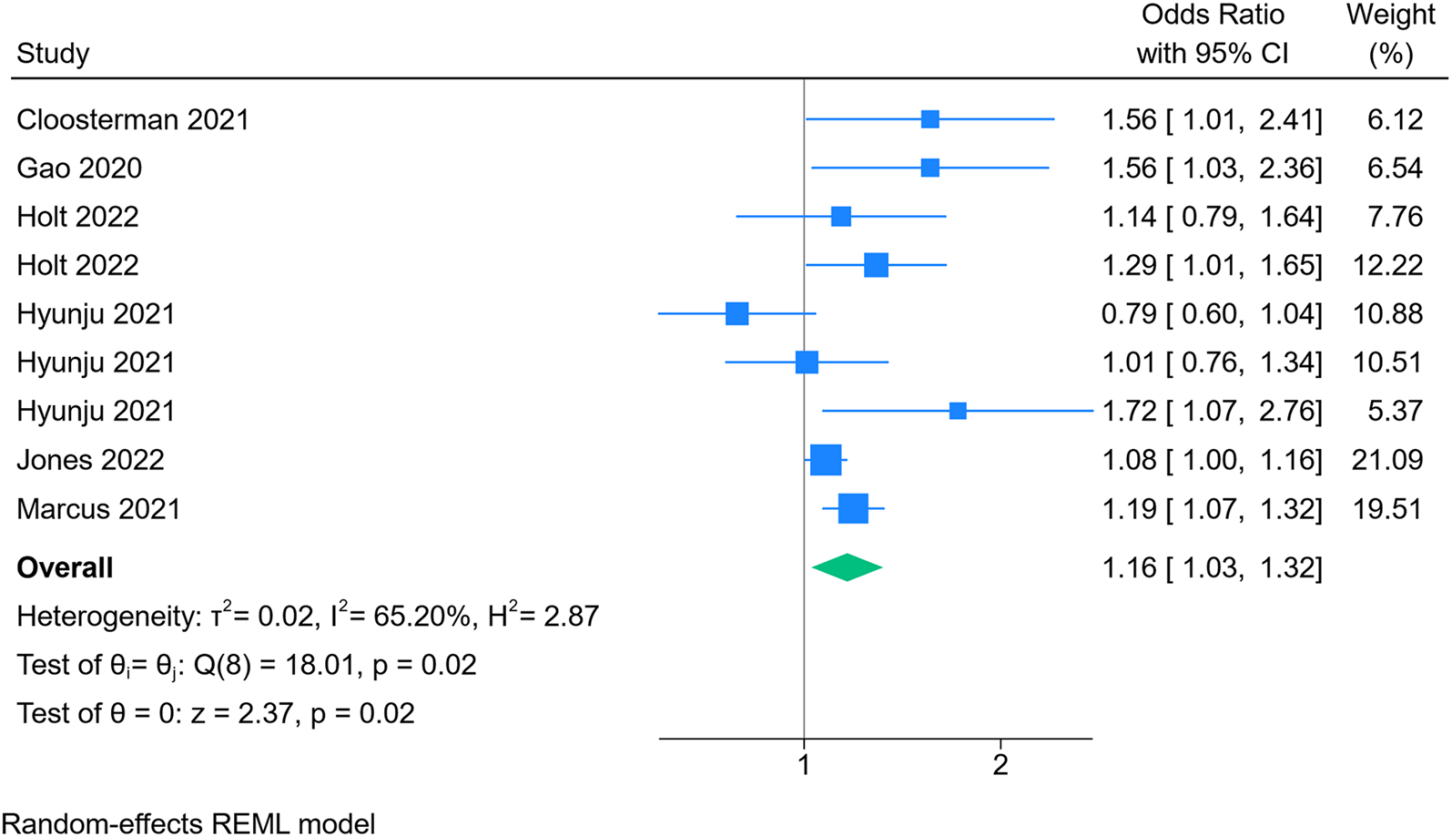
Results of meta-analysis for risk of COVID-19 acquisition

The results of our meta-analysis showed that participants with poor sleep quality showed a 16% increase regarding the risk of COVID-19 acquisition (OR 1.16; 95% CI 1.03, 1.32; *I*^2^ = 65.2%, *p* = 0.02; Fig. 2). The results of our sensitivity analysis based on leave one out are available in Additional file 1: Fig. S2.

### Sleep quality and COVID-19 disease severity

**Fig. 3:**
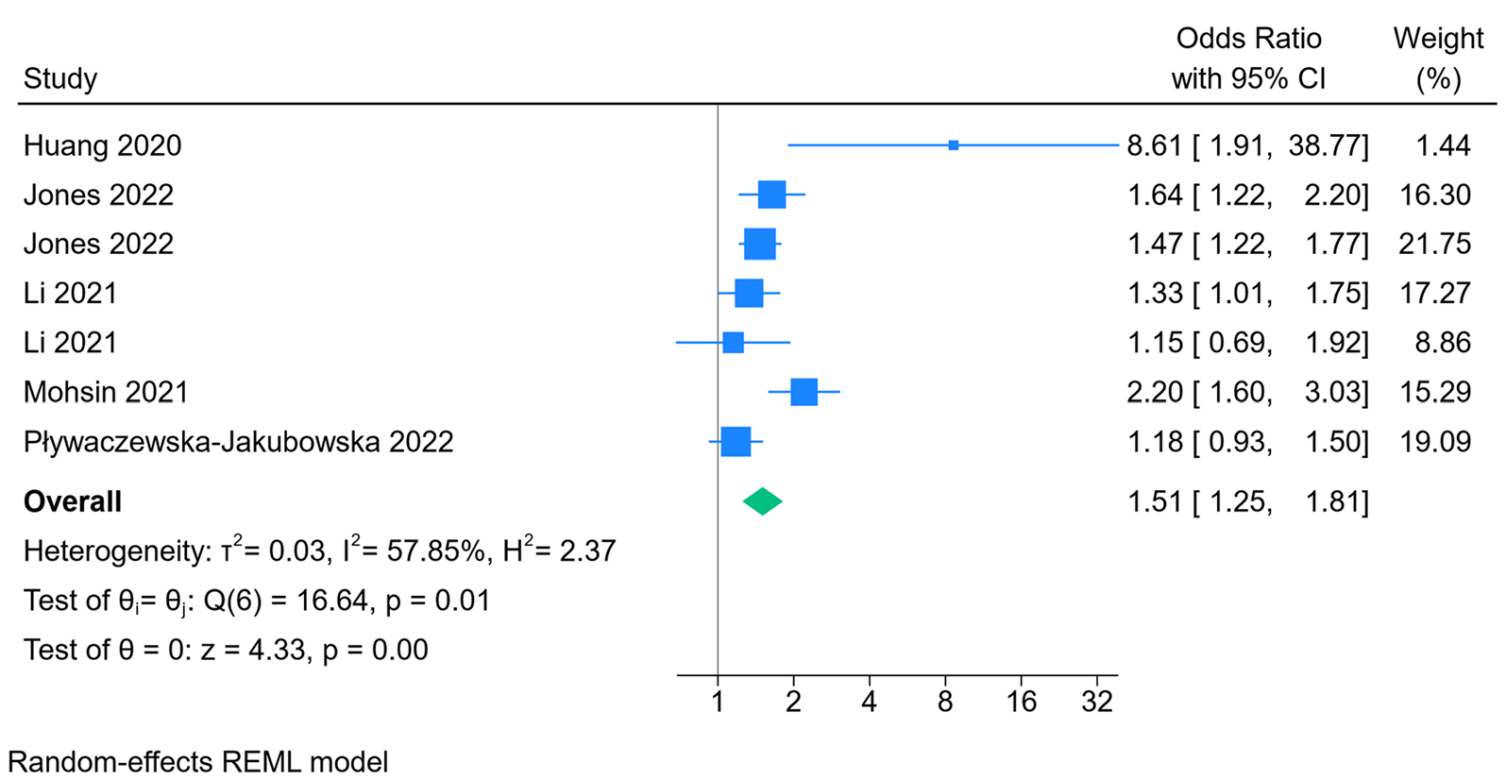
Results of meta-analysis for primary composite outcome

**Fig. 4:**
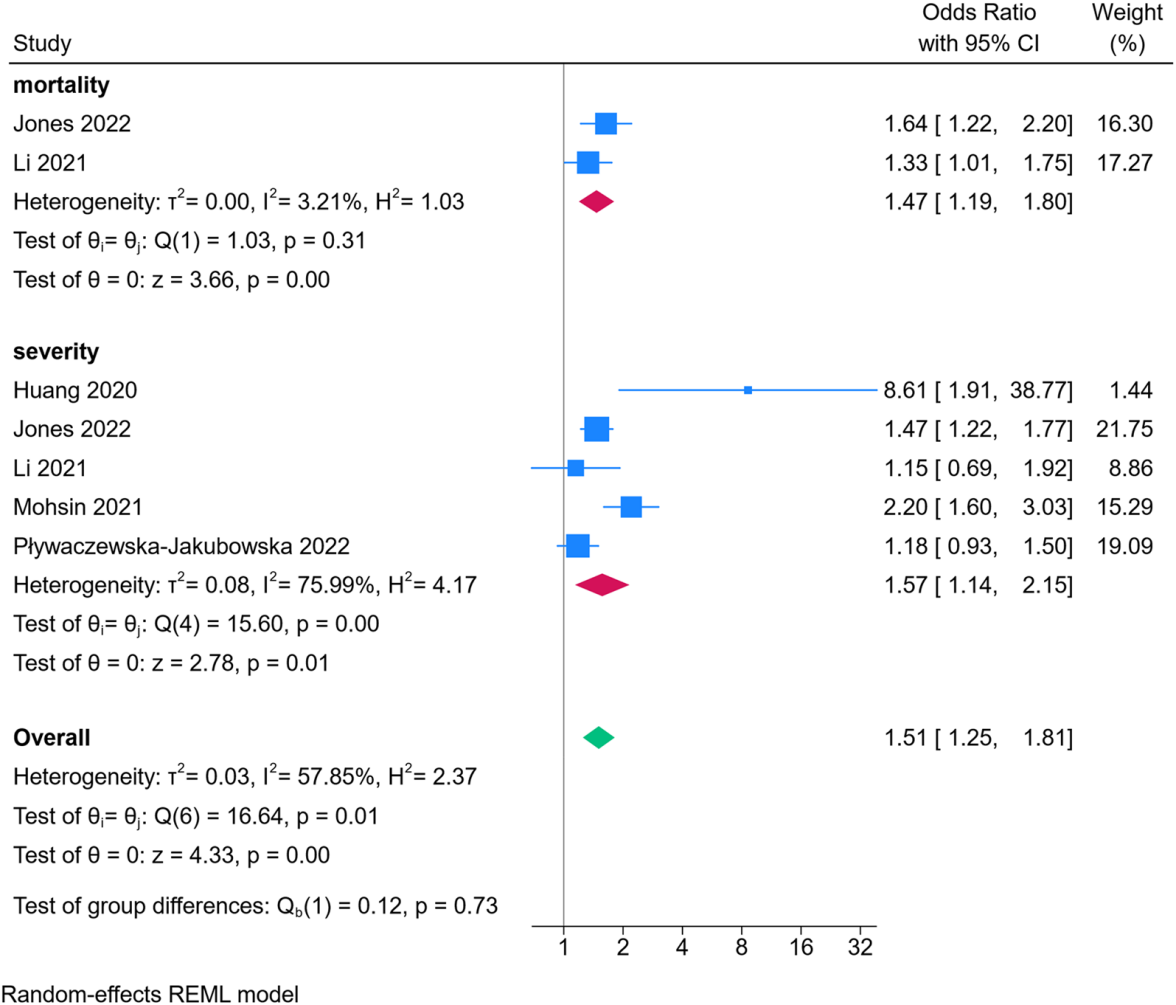
Results of subgroup analysis for primary composite outcome

Our results showed that participants with poor sleep quality showed a 51% increase in the incidence of primary composite outcome (OR 1.51; 95% CI 1.25, 1.81; *I*^2^ = 57.85%, *p* < 0.001; Fig. 3). The result of our subgroup analysis also showed significantly increased risk of mortality (RR 0.67; 95% CI 0.50, 0.90; *I*^2^ = 31%, *p* = 0.008; Fig. 1), and disease severity (OR 1.47; 95% CI 1.19, 1.80; *I*^2^ = 3.21%, *p* < 0.001; Fig. 4) when comparing poor sleep group to those with good sleep quality. The results of our sensitivity analysis based on leave one out are available in Additional file 1: Fig. S2. Only one study reported the hazard ratio of COVID-19 mortality among different quartiles of sleep disturbances [15]. The results of their study showed compared to those in poor sleep quartile, participants in good and moderate group had a HR of 0.80 (95% CI 0.68, 0.95), and 0.83 (95% CI 0.70, 0.98), respectively.

## Discussion

To our knowledge, this study is the first systematic review and meta-analysis which revealed the significant role of good sleep quality in the reduction of both the severity of COVID-19 and the risk of COVID-19 acquisition.

### Impact of sleep quality on COVID-19 infection risk

Our review revealed a significant body of evidence suggesting that poor sleep quality is associated with an increased risk of COVID-19 infection. Several mechanisms may explain this relationship. First, sleep deprivation and disturbances have been shown to compromise immune function, impairing the body's ability to mount an effective defense against viral pathogens [6, 8]. Sleep is essential for immune cell development, cytokine production, and antibody response, which play crucial roles against infections [20], meaning that disruptions in sleep can lead to dysregulation of immune cells and cytokines, making individuals more susceptible to viral infections, including SARS-CoV-2 [28].

Second, inadequate sleep is often accompanied by other risk factors for COVID-19, such as obesity, diabetes, cardiovascular disease, and respiratory disorders [29–31]. These comorbidities have been identified as independent risk factors for severe COVID-19 outcomes [32–34]. Sleep disturbances contribute to the development and exacerbation of these underlying health conditions, further increasing the vulnerability to severe infection [35].

Lastly, Chronic inflammation often seen in individuals with poor sleep quality can also promote a pro-inflammatory environment that facilitates viral replication and worsens disease outcomes [36].

Numerous studies have revealed that lack of sleep is a potential risk factor for COVID-19 infection, a randomized clinical trial done by Gao et al. indicates that 30.5% of patients diagnosed with COVID-19 had sleep deprivation but only 14.8% of healthy individuals had experienced sleep disturbance, which was significantly higher in case group (*p* = 0.001) [18]. Similarly, studies have released that 1-h longer sleep can reduce the risk of COVID infection up to 12% and chronic sleep disorders can increase the risk of different respiratory infections including COVID-19 and average sleep hours in symptoms free cases were significantly higher than COVID-19 patients [21, 22, 24].

It is worth noting that the studies included in our review primarily relied on self-reported measures of sleep quality, such as questionnaires and surveys. Objective measures, such as polysomnography or actigraphy, were less commonly utilized. The reliance on self-reported measures may introduce potential bias due to recall errors and subjective interpretations of sleep quality [37]. Future research should consider incorporating more objective measures of sleep quality to enhance the accuracy of findings.

### Impact of sleep quality on COVID-19 disease severity

In addition to its role in increasing the infection risk, lower sleep quality may also influence the severity of COVID-19 disease [4]. Our study revealed that individuals with poor sleep quality are more likely to experience severe COVID-19 outcomes, as well as increased mortality. This finding is supported by the fact that poor sleep quality compromises both the innate and the adaptive immune system, increasing the risk of infection and reducing the efficacy of vaccines, respectively [7]. Sleep disturbances have also been associated with an increased risk of developing acute respiratory distress syndrome (ARDS), a life-threatening complication of COVID-19 characterized by severe lung inflammation and compromised respiratory function [38], as well as impaired respiratory function, reduced lung capacity, and exacerbation of underlying respiratory conditions; all of which may contribute to the development of severe respiratory complications in COVID-19 patients [4, 39].

Sleep deprivation and poor sleep quality can also lead to alterations in immune cell activity and impaired cytokine regulation, resulting in an exaggerated inflammatory response and cytokine storm observed in some severe cases of COVID-19 [6, 40].

Elise et al. also noted that sleep deprivation may be an indicator of psychological complications, which can increase the risk of severe COVID-19 outcomes.

Lack of sleep plays an important role in disease severity as shown in a study done by Haung et al. COVID severity increases with decreased sleep status as is 8 times higher in patients with lack of sleep [20]. In contrast, another study done by Pkywaczewska-Jakubowska et al. has not reported a significant difference in sleep disturbance or insomnia between various stages of COVID-19 infection [26]. It has been reported that Sleep deprivation is associated with higher rates of mortality and need for hospitalization among COVID-19 patients [23].

Poor sleep quality also appears to be more common among women[41], making them more vulnerable to severe COVID-19 complications, as evident in Pkywaczewska-Jakubowsk et al. study [26]. Although this was not evaluated in any other included study and not enough studies have been included to analyze the effects of gender.

### Implications for public health and clinical practice

The findings of this systematic review have important implications for public health and clinical practice. First, promoting healthy sleep practices and addressing sleep disturbances should be considered as part of comprehensive COVID-19 prevention strategies [42]. Public health campaigns should emphasize the importance of adequate sleep duration, sleep hygiene practices, and stress management techniques to improve sleep quality [43, 44] to help strengthen the immune system and reduce the risk of COVID-19 infection [45].

Incorporating sleep assessment as part of routine clinical evaluations may also help identify individuals at higher risk for severe disease outcomes. Interventions targeting sleep quality, such as cognitive-behavioral therapy for insomnia (CBT-I), can subsequently be implemented on these individuals an adjunctive treatment to improve clinical outcomes in COVID-19 patients [46, 47]. Additionally, healthcare providers should prioritize sleep-related comorbidities, such as obesity, diabetes, and cardiovascular diseases, in the management of COVID-19 patients, as these conditions may worsen the impact of poor sleep quality on disease severity.

## Limitations and future directions

While this systematic review provides valuable insights into the relationship between sleep quality and COVID-19 outcomes, several limitations should be acknowledged. Firstly, most of the included studies were observational in nature, limiting the ability to establish causal relationships between sleep quality and COVID-19 outcomes. Future prospective cohort studies and randomized controlled trials are needed to strengthen the evidence base and establish a causal link. Secondly, heterogeneity in the assessment of sleep quality and COVID-19 outcomes across studies may have influenced the comparability of results. The use of standardized sleep quality measures and consistent definitions of COVID-19 outcomes would enhance the comparability and generalizability of findings across studies.

## Conclusion

This study highlights the potential impact of sleep quality as a risk factor for COVID-19 acquisition and disease severity. Poor sleep quality is associated with an increased risk of COVID-19 infection and a higher likelihood of experiencing severe disease outcomes. Promoting healthy sleep practices, addressing sleep disturbances, and considering sleep-related comorbidities in COVID-19 management strategies have the potential to improve prevention efforts and enhance patient outcomes. Future research should focus on prospective studies and interventions targeting sleep quality to further elucidate the causal relationship and develop targeted interventions for individuals at risk.

### Supplementary Information

**Additional file 1: Table S1.** PRISMA 2020 checklist. **Table S2.** Search strategies for online databases. **Figure S1. **Quality assessment. **Figure S2. **Results of sensitivity analysis for a) COVID-19 Infection Risk; and b) COVID-19 Disease Severity.

## Data Availability

All data have been presented in the manuscript.
